# Early Detection of Common Skin Diseases, Including Leprosy: Development and Validation of an Awareness Questionnaire

**DOI:** 10.3389/ijph.2025.1607938

**Published:** 2025-06-10

**Authors:** Ulfah Abqari, Muhammad Atoillah Isfandiari, Yudhy Dharmawan, Nadhila Beladina, Janet Matani, Mursyidul Ibad, Dian Kusuma, Feby Dwirahmadi, Jan Hendrik Richardus, Ida J. Korfage

**Affiliations:** ^1^ Department of Public Health, Erasmus MC, University Medical Center Rotterdam, Rotterdam, Netherlands; ^2^ NLR Indonesia, Jakarta, Indonesia; ^3^ Faculty of Public Health, Universitas Airlangga, Surabaya, Indonesia; ^4^ Faculty of Public Health, Universitas Diponegoro, Semarang, Indonesia; ^5^ Faculty of Medicine, Public Health, and Nursing, Universitas Gadjah Mada, Yogyakarta, Indonesia; ^6^ Faculty of Public Health, Universitas Nahdatul Ulama Surabaya, Surabaya, Indonesia; ^7^ Health Services Research and Management Department, School of Health & Psychological Sciences, City University of London, London, United Kingdom; ^8^ School of Medicine and Dentistry for the Centre for Environment and Population Health, Griffith University, Nathan, QLD, Australia

**Keywords:** questionnaire, validity, awareness, early detection, skin diseases

## Abstract

**Objectives:**

Skin diseases account for 1.79% of the global disease burden, though their impact may be underreported due to limited research. Raising awareness about early detection is essential, but tools to measure this are scarce. This study aimed to develop and validate a questionnaire assessing community awareness and early detection of common skin diseases, including leprosy, in Indonesia.

**Methods:**

The questionnaire was drafted in English, translated into Bahasa Indonesia, and tested for face validity, content validity, and internal consistency. A pilot test with 25 participants and a field test with 680 participants were conducted. Results were analyzed using Cronbach alpha and descriptive methods.

**Results:**

The final questionnaire comprised 17 questions on skin disease: common knowledge, intentions on prevention and health-seeking behavior. Validity and internal consistency were confirmed during pilot testing, and no participants in the field test reported confusion. The Cronbach alpha score exceeded 0.70, confirming strong internal consistency.

**Conclusion:**

This validated questionnaire can assess public awareness of early skin disease detection. It is available for international adaptation and may help improve early detection and prevention in Indonesia’s healthcare system.

## Introduction

Skin diseases represent a significant global health challenge. According to the Global Burden of Disease (GBD) study, skin and subcutaneous diseases are the fourth most prevalent group of diseases in the world, with a rise in incidence of 46.8% in the past three decades [1, 2]. Collectively, skin and subcutaneous diseases are responsible for 1.79% of the total global disease burden [3]. Skin diseases are diverse, including common skin diseases, such as eczema, and complex systemic illnesses, such as lupus erythematous, HIV/AIDS-related skin diseases, and skin-related Neglected Tropical Diseases (NTDs) such as leprosy [4].

The available data of GBD shows an underestimation of the global burden of skin diseases due to the lack of research. Furthermore, there are challenges in dealing with skin diseases, such as inadequately trained healthcare workers, shortage of programs and facilities in case finding and treatment, and lack of skin disease-related knowledge and awareness in the community [3, 5, 6]. Inadequate knowledge and awareness in the community are considered to be major drivers of stigma towards skin diseases [7, 8]. Leprosy is an important example of a skin-related disease in which unfavorable attitudes in communities are caused by lack of knowledge [9]. Stigma and prejudice against leprosy in a community hinder case finding and treatment, often leading to health and social problems [10, 11]. Inability to timely identify skin diseases in general and leprosy in particular causes delay in detection and treatment [12]. Treatment delay in turn can lead to increased risk of physical disability and reduced quality of life [13–16].

Knowing the level of awareness of skin diseases in a community is essential for developing effective health promotion strategies and interventions to improve early detection we could not identify in literature any specific tools or instruments in the public health (community) domain for measuring such a level of awareness. This study aims to develop and validate a questionnaire for assessing community awareness and early detection of common skin diseases, including leprosy, in Indonesia.

## Methods

### Ethics Statement

Ethical clearance was given by the Faculty of Public Health, Universitas Airlangga, Surabaya, Indonesia (certificate number 160/EA/KEPK/2023). All interview participants provided informed consent (signed or thumb-stamped). Participants received a token of appreciation (e.g., 1 L cooking oil) after the interview. We guarantee confidentiality of data content provided by the participants, e.g., by using only code/randomization number in the database.

### Study Design

**FIGURE 1:**
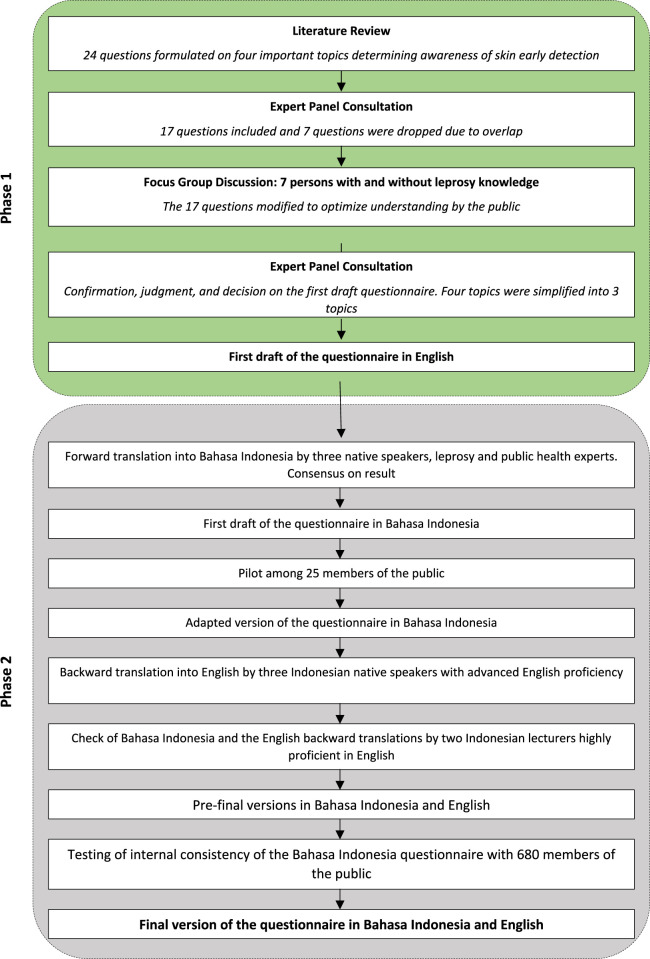
Flowchart of the skin diseases awareness questionnaire development process and its evaluation (Skin Disease Awareness Questionnaire Development Study, Pamekasan, Indonesia, 2023).

A draft questionnaire was developed, translated, and validated. The study design comprised of a pilot test with 25 participants and a final field test involving 680 participants. We conducted repeated tests for face validity, content validity, and internal consistency. The study had two phases as depicted in the flowchart (Figure 1).

### Phase 1: Development of the First Draft Questionnaire (English Version/Original)

Firstly, a draft questionnaire was developed, guided by a literature review of relevant skin problems, early detection awareness (i.e., skin signs and or symptoms present in the areas of common skin diseases knowledge), skin complaint recognition, risk perception, and intention to seek healthcare. We used PubMed database and Google Scholar articles to search for existing instruments that measure the perception of skin disease risk, knowledge of common skin diseases, the recognition of skin complaints, and intention to seek healthcare, with no limits on the year of publication. Keywords used were: “skin disease,” “common,” “knowledge,” “skin complaints,” “risk,” “perception,” “questionnaire,” “scale,” “awareness,” “recognition,” “intention,” “health,” “seek.” The first draft yielded 4 parts with 24 questions from the included articles.

In the next step, expert panel consultation was conducted to reformulate and consider item relevancy. Focus Group Discussion were held with persons affected by leprosy, persons with leprosy expertise (academicians and healthcare workers), including two persons with a history of leprosy or eczema to optimize public understanding. During the Focus Group Discussion, the initial questionnaire which was comprised four was observed and considered there was substantial overlap between the second and third part. As a result, these parts were consolidated into a single section. Of the initial 24 questions, 7 were dropped due to overlap and several modifications were made on the remaining 17 questions. Some words in the question sentences were also altered to enhance the level of understanding of respondents (i.e., changing the question that originally formulated as: *“Do you think the use of protective such as sunscreen, hats, caps, proper clothes that protects sunlight, is necessary to prevent the skin disease?”* to a more direct question addressing actual behavior: *“Do you protect yourself against skin disease, for instance by using soap, hand sanitizer or sunscreen? “*). Details about the content explanations are described in Table 1.

**TABLE 1:** Description of the questionnaire on awareness of skin disease (Skin Disease Awareness Questionnaire Development Study, Pamekasan, Indonesia, 2023).

Content	Description
Part 1: Common skin diseases knowledge	Questions were designed to evaluate the awareness about the occurrence of common skin diseases in the community; not only just the name of a skin disease but also the characteristics of certain symptoms and measures to prevent and cure the disease. Information about skin disease knowledge in the community is essential for planning future public health interventions [17–19]
Part 2: Skin check intention and disease prevention	This part contains questions to collect information about common skin complaints within the community and their ability to recognize each of these skin conditions [17, 19]. Utilizing the Protection Motivation Theory (PMT), this second part of questionnaire also includes questions intended to evaluate the perception and behaviors of the community in preventing skin diseases. This part will subsequently be used to assess whether the perceived need of participants for skin disease prevention significantly influences their health behaviors, particularly their adaptive response and management strategies in coping with, facilitating, or preventing the diseases [20–23]. The findings will be employed to design an educational program for the community
Part 3: Intention to seek health	We used the integrated model of Behavioral Change Model. It is a framework to develop the questions to further understand the predisposing, information, awareness, motivation, as well as barriers that result the actions of health seeking [24–27]

### Phase 2: Translation, Validation, and Internal Consistency of the Questionnaire

We first translated the questionnaire from English into Bahasa Indonesia. This was done by three native translators fluent in English with knowledge about leprosy, common skin diseases, and public health (UA, MAI, and YD). They each independently translated the questionnaire into Bahasa Indonesia, then identified any differences and discussed and resolved these through a consensus meeting. This led to the first Indonesian draft of the questionnaire.

Next, this draft was piloted in face-to-face interviews for content, face validity, and internal consistency among 25 individuals randomly selected public including three local key informants in Batumarmar subdistrict, Pamekasan District. We checked whether terms in the local Madurese language were understood in the same way as the terms used in Bahasa Indonesia. Key informants also recommended word options. Several adjustments were made to the local language, such as changing the word for *sunscreen* to the word *handbody* or *body lotion* because of the familiarity in the community. Several symptoms mentioned in the list of leprosy and other common skin diseases were reduced to the main symptoms that are commonly used in daily conversation, considering that many people have a low health literacy. Unless respondents could mention specific symptoms, an open answer column was applied. The draft was later assessed on language, grammar, and consistency. The pilot study resulted to an adapted version of the questionnaire in Bahasa Indonesia.

After completion of this adapted version, it was backward translated by three native speakers having advanced English proficiency (NB, JM, UA), leading to a second draft of the questionnaire in English. Both the adapted version in Bahasa Indonesia and the second draft in English were reviewed by two Indonesian lecturers (DK and FD) highly proficient in English. Subsequently, the face and content validity and internal consistency of the Bahasa Indonesia version of the questionnaire were assessed among 680 participants in Pamekasan District. This resulted in the final version of the questionnaire in Bahasa Indonesia. The final versions of the questionnaire in Bahasa Indonesia and English are given in the Supplementary Files S1, S2 respectively.

### Study Area and Population

The entire study was conducted in Pamekasan district, East Java Province, Indonesia. Study participants included experts in healthcare and behavioral studies, persons affected by leprosy and eczema representing former skin disease patients, academicians, members of the public, healthcare workers, and local key informants, see Table 2. Participants for the pilot study and validity test of the questionnaire in Bahasa Indonesia had the following eligibility criteria: 1) Residents living in Pamekasan District; 2) Age between 18–65 years; 3) Willing to participate; 4) Able to provide informed consent; and 5) Understand and communicate in Bahasa Indonesia and Madura language.

**TABLE 2:** General characteristics of the study population per study element (Skin Disease Awareness Questionnaire Development Study, Pamekasan, Indonesia, 2023).

Phase	Study element	Population	N	Gender
1	Expert panel consultations	Research Supervisors	3	M: 2 F: 1
1	Focus Group Discussion	Persons affected by leprosy and eczema, persons with leprosy knowledge including healthcare workers	7	M: 1 F: 6
2	Forward translation of the original English version into Bahasa Indonesia	Native speakers and experts in leprosy and public health	3	M:2 F: 1
2	Questionnaire pilot: face and content validity, and internal consistency (Bahasa Indonesia version)	Members of the public including local key informants	25	M: 10 F: 15
2	Backward translation into English	Research officers with advanced English proficiency	3	M: 0 F: 3
2	Check of both Bahasa Indonesia and English questionnaires	Indonesian lecturers teaching with high proficiency in English	2	M: 2 F: 0
2	Interview to test again the face and content validity, and internal consistency	Members of the public	680	M: 226 F: 454

### Data Analysis

The interviewers for the pilot and the field study were recruited among experienced health workers, trained to use the questionnaire and collect data through interviews with members of the public. The following demographic information was collected: gender, age, education level, type of occupation and type of residency (urban or rural). Data were analyzed using SPSS 21. We performed descriptive and analytic statistics. Validity and internal consistency tests were conducted on data of the pilot stage of 25 participants and of the field study of 680 participants. Face and content validity were determined to qualitatively measure the validity of the questionnaire by evaluating the response, expression, and explanation of participants [28]. The internal consistency was analyzed statistically using Cronbach-alpha [29].

## Results

### Characteristics of Participants

Table 2 provides an overview of the general characteristics of the study population per study element. Demographic information on the study participants in pilot (n = 25) and field (n = 680) tests is given in Table 3.

**TABLE 3:** Characteristics of participants in the pilot test and field test (Skin Disease Awareness Questionnaire Development Study, Pamekasan, Indonesia, 2023).

		Pilot test		Field test	
		Participants (n = 25)	%	Participants (n = 680)	%
Age	Years	35.88 ± 8.88		37.89 ± 12.38	
Gender	Male Female	10	40	226	33.24
		15	60	454	66.76
Education	No formal education Elementary School Junior High School Senior High School Tertiary degree	2	8	1	0.15
		5	20	192	28.24
		2	8	119	17.50
		4	16	255	37.50
		12	48	113	16.62
Occupational	Government officials/state-owned enterprise/police/army Self-employed Private sector employee Farmer/fisher Laborer/driver/household Assistant Unemployed Others	0	0	19	2.79
		3	12	176	25.88
		12	48	59	8.68
		0	0	100	14.71
		0	0	34	5.00
		9 1	36 4	230 62	33.82 9.12

### Face and Content Validity Tests

During the pilot study, three out of 25 participants asked for further explanation regarding the questions. Otherwise, during the pilot study and the field study (Table 4), participants did not express confusion or uncertainty about the questions. As all questions were noted as comprehensible, face validity was considered to be adequate.

**TABLE 4:** Face and content validity results from the pilot study and the field study (Skin Disease Awareness Questionnaire Development Study, Pamekasan, Indonesia, 2023).

	Pilot study (n = 25)	Field study (n = 680)
Timeline	July 2023	August 28th – October 27th^,^ 2023
Face validity	3 participants asked for the meaning of certain words. The interviewers needed to change the words in local terms. All other participants answered the questions without confusion There were no uncertainties or time lag of the respondents answering the questions	All participants answered the questions well. They expressed no confusion and uncertainty through their body language. Only a few people needed extra time to recall the name of a skin disease or to mention symptoms. They understood the questions well
Content validity	All respondents expressed that the questionnaire is easy to understand. they could easily share the common issues of skin diseases they know. They also shared what influenced their decision to seek health or not	All respondents indicated that the questionnaire is easy to understand because the terms are well-known. They were happy with the questionnaire because it raises important topics for them that they should know and be more aware of

### Internal Consistency

Internal consistency is shown in Table 5. All scores were more than 0.70, indicating strong internal consistency of the topics [29].

**TABLE 5:** Results of internal consistency testing of the questionnaire by the Cronbach Alpha test (Skin Disease Awareness Questionnaire Development Study, Pamekasan, Indonesia, 2023).

Part	Cronbach alpha score	
	Pilot study (n = 25)	Field study (n = 680)
A	0.784	0.823
B	0.760	0.720
C	0.731	0.738
Total Questions	0.863	0.789

## Discussion

Despite skin diseases being very common, its burden is underestimated considerably due to several factors, including lack of knowledge and awareness within the community [3, 5, 6]. Understanding the extent of community awareness of skin diseases is key for developing effective health promotion strategies and interventions for the prevention of skin diseases. This study is the first to newly develop a questionnaire to measure the level of awareness in the community of common skin diseases. We employed several comprehensive methods including literature review, focus group discussions, and expert panel consultation. The use of the questionnaire in this study proved to be highly effective in fulfilling its objectives. It was designed to assess the participants’ common knowledge of skin diseases, their intentions regarding checking and preventing such diseases, and their willingness to seek health services. The large study population (n = 680) allowed for a comprehensive evaluation of the tool’s utility. The internal consistency (Cronbach’s alpha >0.70 across all sections) further underscores the reliability of the questionnaire in measuring the intended constructs. Additionally, participants’ feedback during the pilot and field tests indicated that the questions were clear and easy to understand, thereby affirming the questionnaire’s face and content validity. The findings suggest that the questionnaire successfully achieved its purpose and holds potential for broader application in similar public health contexts.

The procedure for developing the questionnaire adhered to established methodologies employed previously in the development of questionnaires for skin disease programmes [30, 31]. In addition to the linguistic and content validation, a face validity test was also employed, taking into account the perceived stigma that exists among patients with skin conditions [32, 33]. In addition to ensuring the absence of item ambiguity, the employment of face validity serves as a sensitivity test, clarifying that the question items are not judgmental, distressing, or intrusive for the target participants [34].

The questionnaire was in the Madurese language, the local language of the respondents. An effort was made to use terms in the questionnaire that could be understood by the Madurese respondents. They understood the questionnaire well, thereby demonstrating its face validity and content validity. The Internal consistency showed that public awareness of the need for early detection of skin diseases in general and leprosy in particular can be assessed by the questions in the questionnaire.

We proved the questionnaire to be a valid and reliable instrument to assess public awareness of the need for early detection of skin diseases. This questionnaire is important to avoid delay in the detection of skin diseases, including leprosy. Through awareness people can seek for timely diagnosis and treatment at health services. Delay in diagnosis and treatment can lead to severe complications. For instance, the delay of leprosy diagnosis can lead to irreversible disability [12]. The questionnaire can be used as health promotion tool for health staff in skin disease programs to increase public awareness and detect skin diseases in time and would be of benefit to the Indonesian health services.

This study is the first to report the development of a questionnaire to assess public awareness of the need for early detection of skin diseases in general and leprosy in particular. The strength of the study is that it applies robust questionnaire development methodology and the findings from this study offer critical insights into the level of awareness and behavioural intentions related to skin diseases in a community setting. These insights can guide targeted health promotion strategies, early detection programs, and skin health interventions. A limitation is that we could not perform a complete validation test including construct and criterion validity due to the lack of a gold standard instrument to assess public awareness of the need for early detection of skin diseases [29]. Before implementing this questionnaire broadly in Indonesia, we recommend to test validity in other areas of Indonesia and adapt it to local dialect or language, because of the substantial variation in cultures and languages in Indonesia. An English version is also available for validation and adaption in international context.

We conclude that the questionnaire holds potential as a tool to enhance awareness and mitigating delays in skin diseases detection. We recommend its integration into Indonesian health programs and healthcare facilities.
