# Supplementary material for: Early Detection of Common Skin Diseases, Including Leprosy: Development and Validation of an Awareness Questionnaire
Source: Int J Public Health. 2025 Jun 10;70:1607938. doi: 10.3389/ijph.2025.1607938 (PMC12202976; doi:10.3389/ijph.2025.1607938)
Supplement: Supplementary file 1 [file Table1.docx]

**Supplementary File 1. Awareness Questionnaire of Skin Diseases Early Detection (Bahasa Indonesia)**

| **Identitas Responden Nomor ID peserta** | | |
| --- | --- | --- |
| Nama | : |  |
| Usia | : | tahun |
| Jenis Kelamin | : | - 1. Pria - 2. Wanita |
| Pendidikan | : | - 1. SD/MI - 2. SMP/MTS - 3. SMA/MA - 4. S1/S2/S3 |
| Pekerjaan | : | - 1. PNS/BUMN/Polri/TNI - 2. Wiraswasta - 3. Karyawan Swasta - 4. Petani/Nelayan - 5. Buruh/Sopir/Pembantu RT - 6. Tidak Bekerja - 7. Lain-lain |

| **Pengetahuan tentang penyakit kulit yang umum** |
| --- |

1. Apakah Anda pernah mengalami penyakit kulit?

- Ya (lanjut ke no. 2)
- Tidak (lanjut ke no. 3)
- Tidak yakin (lanjut ke no. 3)

1. (Jika menjawab YA) Bisakah Anda menyebutkan nama penyakit kulit yang pernah Anda alami?
2. (Jika menjawab TIDAK atau TIDAK YAKIN) Bisakah Anda menyebutkan 1 atau 2 nama penyakit kulit yang Anda ketahui?
3. Bisakah Anda menyebutkan gejala penyakit kulit yang Anda sebutkan di pertanyaan no. 2 dan 3? (Beri tanda √ (centang) pada jawaban yang Anda pilih!)

|  | Penyakit kulit 1  (sebutkan .........) | Penyakit kulit 2  (sebutkan .......) | Penyakit kulit 3  (sebutkan .......) | Jika ya, lanjut ke pertanyaan tentang kusta pada no. 8 |
| --- | --- | --- | --- | --- |
| 1. Bercak pada kulit |  |  |  |  |
| 1. Ruam |  |  |  |  |
| 1. Warna tidak merata |  |  |  |  |
| 1. Luka |  |  |  |  |
| 1. Perubahan warna kulit |  |  |  |  |
| 1. Perdarahan |  |  |  |  |
| 1. Kulit kering |  |  |  |  |
| 1. Benjol |  |  |  |  |
| 1. Mati rasa |  |  |  |  |
| 1. Nyeri |  |  |  |  |
| 1. Gatal |  |  |  |  |
| 1. Lainnya (sebutkan) .......... |  |  |  |  |

1. Apakah Anda tahu penyebab penyakit kulit yang Anda sebutkan tadi? Jika ya, tolong sebutkan!

|  | Penyakit kulit 1  (sebutkan .........) | Penyakit kulit 2  (sebutkan .......) | Penyakit kulit 3  (sebutkan .......) | Jika ya, lanjut ke pertanyaan tentang kusta pada no. 8 |
| --- | --- | --- | --- | --- |
| Penyebab | - Ya, sebutkan | - Ya, sebutkan | - Ya, sebutkan | - Ya, sebutkan |
|  | - Tidak tahu | - Tidak tahu | - Tidak tahu | - Tidak tahu |

1. Tindakan apa yang Anda lakukan untuk mencegah penyakit kulit? ((Beri tanda √ (centang) pada jawaban yang Anda pilih!) Bisa pilih lebih dari satu jawaban)

|  | Penyakit kulit 1  (sebutkan .........) | Penyakit kulit 2  (sebutkan .......) | Penyakit kulit 3  (sebutkan .......) | Jika ya, lanjut ke pertanyaan tentang kusta pada no. 8 |
| --- | --- | --- | --- | --- |
| 1. Mencegah kontak dengan penderita penyakit kulit menular |  |  |  |  |
| 1. Menggunakan pelindung untuk meminimalisir paparan sinar matahari |  |  |  |  |
| 1. Menjaga kebersihan |  |  |  |  |
| 1. Lainnya (sebutkan) ..................................... |  |  |  |  |

1. Apa yang dapat dilakukan seseorang untuk menyembuhkan diri dari penyakit kulit? ((Beri tanda √ (centang) pada jawaban yang Anda pilih!) Bisa pilih lebih dari satu jawaban)

|  | Penyakit kulit 1  (sebutkan .........) | Penyakit kulit 2  (sebutkan .......) | Penyakit kulit 3  (sebutkan .......) | Jika ya, lanjut ke pertanyaan tentang kusta pada no. 8 |
| --- | --- | --- | --- | --- |
| 1. Periksa ke dokter |  |  |  |  |
| 1. Diobati sendiri |  |  |  |  |
| 1. Pergi ke pengobatan tradisional |  |  |  |  |
| 1. Dibersihkan dengan air |  |  |  |  |
| 1. Ditunggu hingga sembuh |  |  |  |  |
| 1. Tidak melakukan apapun |  |  |  |  |
| 1. Lainnya (sebutkan) .................................. |  |  |  |  |

1. Apakah Anda pernah mendengar tentang penyakit kusta?
   - - - 1. Tidak, lanjut ke bagian berikutnya
       - 2. Ya, kembali ke pertanyaan no. 4, 5, 6 dan 7 tentang penyakit kusta

| **Upaya Pemeriksaan Kulit dan Pencegahan Penyakit** |
| --- |

1. Apakah Anda pernah memeriksa kemungkinan adanya tanda penyakit kulit pada tubuh Anda, misalnya ketika berpakaian?
   - - - 1. Ya
       - 2. Tidak
2. Jika ya, seberapa sering Anda memeriksanya? (bisa pilih lebih dari satu jawaban)

- 1. Secara teratur, ketika saya mandi, bercermin, atau berpakaian
- 2. Hanya ketika saya tiba-tiba merasakan gejalanya seperti gatal
- 3. Setelah melihat anggota keluarga atau orang sekitar saya mengeluhkan penyakit kulit
- 4. Ketika saya diberi tahu orang lain
- 5. Lainnya, sebutkan……………

1. Apakah Anda melindungi diri dari penyakit kulit, misalnya dengan sabun, desinfektan tangan (hand sanitizier), atau krim anti UV (sunscreen)?

- 1. Ya, setiap saat/sering
- 2. Ya, kadang-kadang
- 3. Tidak, tidak pernah

1. Jika “Tidak, tidak pernah” atau “Ya, kadang-kadang”, apa alasan Anda? (bisa pilih lebih dari satu jawaban)

- 1. Saya merasa tidak nyaman
- 2. Saya merasa tidak perlu
- 3. Saya sering lupa
- 5. Lainnya (sebutkan)

| **Niat mencari layanan kesehatan** |
| --- |

1. Jika Anda mengalami keluhan pada kulit, apa yang akan Anda lakukan?

- 1. Saya tidak melakukan apa-apa
- 2. Saya akan mengobatinya sendiri
- 3. Saya akan pergi ke fasilitas kesehatan, seperti Puskesmas
- 4. Saya akan pergi ke pengobatan tradisional
- 5. Lainnya, sebutkan

1. Apa alasan yang menjadi pertimbangan Anda saat menjawab pilihan pada no. 13? (bisa pilih lebih dari satu jawaban dan menambahkan alasan lain jika ada)

- 1. Saya takut jika didiagnosis penyakit tertentu
- 2. Was-was / gelisah
- 3. Malu
- 4. Tidak diizinkan anggota keluarga (misalnya: oleh pasangan(suami/istri) atau orangtua)
- 5. Tidak ada biaya
- 6. Belum punya asuransi kesehatan
- 7. Lainnya (sebutkan)

1. Jika Anda memiliki keluhan pada kulit dan pergi ke fasilitas kesehatan, kapan Anda akan melakukannya?

- 1. Segera setelah melihat tanda atau merasakan keluhan kulit
- 2. Saya akan menunggunya terlebih dahulu
- 3. Setelah mendapat saran dari orang terdekat
- 4. Saya tidak tahu
- 5. Lainnya, sebutkan

1. Apa alasan yang menjadi pertimbangan Anda saat menjawab pilihan pada no. 15?? (bisa pilih lebih dari satu jawaban)

- 1. Agar membaik
- 2. Agar tidak semakin memburuk
- 3. Permintaan teman / keluarga saya
- 4. Lainnya (sebutkan)

1. Apakah Anda menyarankan orang lain yang mengalami keluhan kulit untuk memeriksakan dirinya ke fasilitas kesehatan?

- 1. Ya
- 2. Tidak
