# Supplementary material for: Early Detection of Common Skin Diseases, Including Leprosy: Development and Validation of an Awareness Questionnaire
Source: Int J Public Health. 2025 Jun 10;70:1607938. doi: 10.3389/ijph.2025.1607938 (PMC12202976; doi:10.3389/ijph.2025.1607938)
Supplement: Supplementary file 2 [file Table2.docx]

**Supplementary 2. Awareness Questionnaire of Skin Diseases Early Detection (English)**

| **Identity of Respondent Subject ID Number** | | |
| --- | --- | --- |
| Name | : |  |
| Age | : | years old |
| Gender | : | - 1. Male - 2. Female |
| Educational degree | : | - 1. No having formal education - 2. Elementary school - 3. Junior high school - 4. Senior high school - 5. Bachelor’s degree/Master/PhD |
| Occupation | : | - 1. Government officials/State-owned enterprise/Police/Army - 2. Self-employed - 3. Private sector employee - 4. Farmer/Fisher - 5. Laborer/Driver/Household assistant - 6. Unemployed - 7. Others |

| **Knowledge about Common Skin Diseases** |
| --- |

- - - 1. Have you ever had a skin disease?
- Yes (continue to no. 2)
- No (continue to no. 3)
- Not sure (continue to no. 3)
  - - 1. (If YES) Could you please mention the name of the skin disease(s) you had?
      2. (If NO or NOT SURE) Could you mention the name of 1 or 2 skin disease(s) you know?
      3. Could you mention the signs and symptoms of the skin diseases that you mentioned in questions 2 and 3? (Please give √ mark (check) for the selected answer!)

|  | Skin disease 1  (please specify) | Skin disease 2  (please specify) | Skin disease 3  (please specify) | If the answer is yes for no 8, please continue to the questions about leprosy in the last column in no 4, 5 and 6 |
| --- | --- | --- | --- | --- |
| 1. Skin patches |  |  |  |  |
| 1. Rash |  |  |  |  |
| 1. Uneven skin tone |  |  |  |  |
| 1. Wound |  |  |  |  |
| 1. Skin discoloration |  |  |  |  |
| 1. Bleeding |  |  |  |  |
| 1. Dry skin |  |  |  |  |
| 1. Nodules |  |  |  |  |
| 1. Numbness/lost of sensation |  |  |  |  |
| 1. Pain |  |  |  |  |
| 1. Itch |  |  |  |  |
| 1. Others, please specify .......... |  |  |  |  |

- - - 1. Do you know the cause of the skin disease(s) as mentioned before in question 4? If yes, please specify!

|  | Skin disease 1  (please specify) | Skin disease 2  (please specify) | Skin disease 3  (please specify) | If yes, please continue to the question number 8 about leprosy |
| --- | --- | --- | --- | --- |
| The cause | - Yes, namely | - Yes, namely | - Yes, namely | - Yes, namely |
|  | - Don’t know | - Don’t know | - Don’t know | - Don’t know |

- - - 1. What would you do to prevent the skin disease(s)? (Please give √ mark (check) for the selected answer! You can choose more than one answer)

|  | Skin disease 1  (please specify) | Skin disease 2  (please specify) | Skin disease 3  (please specify) | If yes, please continue to the question number 8 about leprosy |
| --- | --- | --- | --- | --- |
| 1. Prevent contact with someone having infectious skin diseases |  |  |  |  |
| 1. Take protective measures to minimize sun exposure |  |  |  |  |
| 1. Maintain hygiene |  |  |  |  |
| 1. Others, please specify .................. |  |  |  |  |

- - - 1. What could someone do to cure the skin disease? (Please give √ mark (check) for the selected answer! You can choose more than one answer)

|  | Skin disease 1  (please specify) | Skin disease 2  (please specify) | Skin disease 3  (please specify) | If yes, please continue to the question number 8 about leprosy |
| --- | --- | --- | --- | --- |
| 1. See a physician |  |  |  |  |
| 1. Self-medicate |  |  |  |  |
| 1. Go to a traditional healer |  |  |  |  |
| 1. Clean with water |  |  |  |  |
| 1. Wait until it heals |  |  |  |  |
| 1. Do nothing |  |  |  |  |
| 1. Others, please specify ...................................... |  |  |  |  |

1. Have you ever heard about leprosy?

- 1. No, please continue to the next section
- 2. Yes, please go back to questions no. 4, 5, 6 and 7 about leprosy

| **Skin Check Intention and Diseases Prevention** |
| --- |

1. Have you ever checked for possible signs of skin diseases on your body, for example when putting on clothes?

- 1. Yes
- 2. No

1. If yes, how often do you check it? (More than one answer can be chosen)

- 1. Regularly, when I am taking a bath, looking in the mirror, or putting on clothes
- 2. Only when I suddenly feel the symptoms, such as itching
- 3. After seeing family members or people around me having skin complaints
- 4. When I am notified by other people
- 5. Others, please specify ………………….

1. Do you protect yourself against skin diseases, for example by using soap, hand sanitizer, or cream for anti-UV (sunscreen)?

- 1. Yes, regularly
- 2. Yes, sometimes
- 3. No, never

1. If “No, never” or “Yes, sometimes”, what is your reason? (more than one answer can be chosen)

- 1. I feel uncomfortable
- 2. I feel unnecessary
- 3. I tend to forget
- 4. Others, please specify

| **Intention to Seek Health Services** |
| --- |

1. If you experience skin complaints, what will you do?

- 1. I will do nothing
- 2. I will self-medicate
- 3. I will go to a health facility, such as Puskesmas
- 4. I will go to a traditional healer
- 5. Others, please specify

1. What reasons did you consider when answering no. 13? (more than one answer can be selected and another reason can be added)

- 1. I am afraid of being diagnosed with a certain disease
- 2. I feel anxious
- 3. I am ashamed
- 4. Not permitted by family members (for example: spouse (husband/wife) or parents)
- 5. There is no budget
- 6. Do not have health insurance
- 7. Others, please specify

1. If you have a skin complaint and decide to go to health facilities, when will you do it?

- 1. Immediately after seeing the signs or feeling the skin complaints
- 2. I will take some time to wait
- 3. After getting advice from a close person(s)
- 4. I don’t know
- 5. Others, please specify

1. What reasons did you consider when answering no. 15? (more than one answer can be selected and another reason can be added)

- 1. To get better
- 2. To prevent getting worse
- 3. My friend/family’s request
- Others, please specify

1. Would you recommend other people who have skin complaints to check at a health facility?

- 1. Yes
- 2. No
